# Progress and Outlook on Electrochemical Sensing of Lung Cancer Biomarkers

**DOI:** 10.3390/molecules29133156

**Published:** 2024-07-02

**Authors:** Rui Zheng, Aochun Wu, Jiyue Li, Zhengfang Tang, Junping Zhang, Mingli Zhang, Zheng Wei

**Affiliations:** 1 The Second School of Clinical Medicine, Henan University of Chinese Medicine, Zhengzhou 450053, China; 2021006008@hactcm.edu.cn (R.Z.); wuaochuneb@126.com (A.W.); 2 Cancer Research Institute, Henan Integrative Medicine Hospital, Zhengzhou 450003, China; zhang120@126.com (M.Z.); weizheng@hactcm.edu.cn (Z.W.); 3 The First School of Clinical Medicine, Henan University of Chinese Medicine, Zhengzhou 450099, China; 18300699023@163.com (J.L.); 2022704312@hactcm.edu.cn (Z.T.)

**Keywords:** voltammetry, microfluidics, nanocomposites, impedance, multiplexing

## Abstract

Electrochemical biosensors have emerged as powerful tools for the ultrasensitive detection of lung cancer biomarkers like carcinoembryonic antigen (CEA), neuron-specific enolase (NSE), and alpha fetoprotein (AFP). This review comprehensively discusses the progress and potential of nanocomposite-based electrochemical biosensors for early lung cancer diagnosis and prognosis. By integrating nanomaterials like graphene, metal nanoparticles, and conducting polymers, these sensors have achieved clinically relevant detection limits in the fg/mL to pg/mL range. We highlight the key role of nanomaterial functionalization in enhancing sensitivity, specificity, and antifouling properties. This review also examines challenges related to reproducibility and clinical translation, emphasizing the need for standardization of fabrication protocols and robust validation studies. With the rapid growth in understanding lung cancer biomarkers and innovations in sensor design, nanocomposite electrochemical biosensors hold immense potential for point-of-care lung cancer screening and personalized therapy guidance. Realizing this goal will require strategic collaboration among material scientists, engineers, and clinicians to address technical and practical hurdles. Overall, this work provides valuable insight for developing next-generation smart diagnostic devices to combat the high mortality of lung cancer.

## 1. Introduction

SCLC is an extremely aggressive subtype, constituting 10–15% of all lung malignancies. It is characterized by rapid doubling time and high metastatic potential. Hence, despite good initial chemosensitivity, the 5-year survival rate for SCLC patients with extensive disease remains dismal at 7% [1]. This highlights the pressing need for the development of new diagnostic strategies that can detect tumors early when localized and curable [2]. Biomarkers are measurable indicators of physiological or pathological processes that have shown great promise for cancer screening [3]. Electrochemical biosensing has emerged as a cutting-edge technology for fast, ultrasensitive, specific, and cost-effective detection of cancer biomarkers, holding immense potential to enable early SCLC diagnosis and therapy [4].

**Figure 1:**
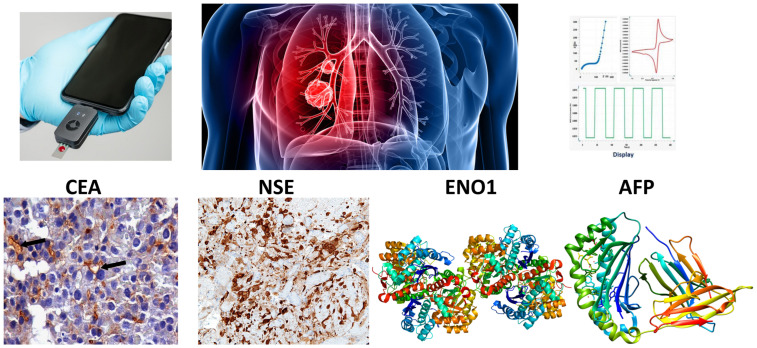
Common biomarkers for SCLC diagnosis.

Serum proteins like CEA and NSE, gene expression markers, namely, alpha enolase (ENO1 and ENO2), as well as glycoprotein AFP, have demonstrated clinical utility as circulating biomarkers for SCLC [5]. CEA is an oncofetal glycoprotein involved in cellular adhesion, highly expressed in endodermal epithelial tumors [6]. Elevated CEA levels often signify metastatic SCLC and hence indicate poor prognosis. NSE is an isoenzyme released by neurons and neuroendocrine cells, routinely employed as a biomarker in the diagnosis, staging, and monitoring of SCLC, particularly when levels exceed 25 ng/mL [7]. The enolase genes ENO1 and ENO2 respectively encode neuron-specific γ and ubiquitous α isoforms of the glycolytic enzyme enolase. The overexpression of ENO1 and loss of ENO2 observed in SCLC tumors promote neoplastic proliferation via mechanisms like DNA hypomethylation and PI3K/AKT pathway activation [8]. AFP is a serum glycoprotein biomarker employed chiefly for germ cell tumors, but emerging evidence supports its diagnostic significance for SCLC too [9]. Quantitating these circulating protein and genetic biomarkers offers a non-invasive liquid biopsy strategy for SCLC screening. Figure 1 shows the common biomarkers for SCLC diagnosis.

The exponential progress in biosensor research has spawned innovative devices that can sensitively and selectively capture cancer biomarkers within minutes. As defined by IUPAC, biosensors are small integrated receptor–transducer devices capable of providing specific quantitative or semi-quantitative analytical data using biological recognition elements. Electrochemical biosensors efficiently translate molecular recognition events at conductive interfaces into readable electrical signals for point-of-care use [10]. Their benefits like rapid responses, low sample volume needs, cost efficiency, potential for miniaturization, and simplicity of mass manufacturing have fueled tremendous growth in biomarker quantification applications [11]. Key components in these devices are electrode substrates providing a conductive platform to immobilize biorecognition elements like antibodies, aptamers, DNA/RNA probes, or enzymes [12]. Specific binding of analyte biomarkers then leads to measurable electronic changes. Depending on transduction modes, measurable parameters are current, potential, impedance, or conductance changes [13]. Common electrochemical techniques employed are amperometry, potentiometry, conductometry, and impedance spectroscopy. Advanced sensor configurations also harness nanomaterials or signal enhancement strategies to lower the limits of detection. Capitalizing on such technology innovations, ultrasensitive detection limits up to fg/mL to ag/mL levels have been achieved for cancer biomarkers using nanostructured sensors [14].

Over the past decade, electrochemical biosensors have made tremendous headway in addressing the limitations of routine immunoassays for cancer diagnosis. Microfluidic multiplexing with graphene and gold nanoparticles has enabled simultaneous measurements of multi-cancer biomarker panels necessary to improve diagnostic specificity [15]. Techniques to concentrate diluted biomarkers in biological fluids mitigate matrix effects and variability challenges. Advances in synthetic antibodies, aptamers, and programmable biorecognition elements enhance stability for reuse, while novel interfaces with biomimetic properties lower fouling and non-specific adsorption [16]. Wearable sweat-based sensors and the integration of lab-on-chip devices with smartphones demonstrate the feasibility of rapid, point-of-care cancer screening [17]. Despite these developments, however, most electrochemical biosensors still remain confined to academic laboratories without extensive clinical translation. Issues related to reproducibility, a lack of standardization of biomarker cutoffs, and the paucity of large-scale validation studies need addressing for future adoption into clinics.

Electrochemical biosensors for cancer diagnostics show immense future capability but greater collaboration across various disciplines and strong industry partnerships is necessary to bridge existing gaps in SCLC biomarker detection technology. With our growing understanding of disease biomarkers, adoption of Quality by Design and mass manufacturing principles, and innovations in application-specific sensor materials and surface chemistries, next-generation point-of-care biosensors can transform the management of aggressive cancers [18]. This review comprehensively discusses recent progress in the electrochemical quantification of critical SCLC biomarkers like CEA, NSE, and AFP. Key aspects include commonly used sensor techniques, the role of nanomaterials, current sensing strategies and performance metrics, biological sample analysis needs, integration and multiplexing solutions, and existing technology barriers, as well as an outlook on the future prospects of electrochemical devices in early diagnosis and screening applications against SCLC.

## 2. Electrochemical Sensing Techniques and Strategies

### 2.1. Basic Principles of Electrochemical Techniques

Electrochemical sensors translate molecular recognition events occurring at electrode–electrolyte interfaces into useful electrical signals for quantitative measurements in a rapid and high-throughput manner. Selectivity arises from the use of specific bioreceptor molecules that only interact with target analytes [19]. The ensuing electronic changes are measured by specialized electrochemical techniques like amperometry, potentiometry, or impedance spectroscopy to obtain sensing parameters.

**Figure 2:**
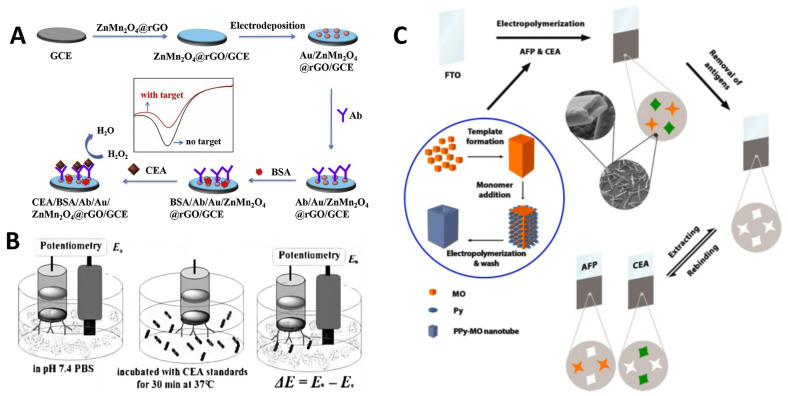
(**A**) Amperometric immunosensor for the detection of CEA using ZnMn_2_O_4_@reduced graphene oxide composite [20]. (**B**) Potentiometrical electrochemical sensor for CEA detection [23]. (**C**) Dual-template rectangular nanotube molecularly imprinted polypyrrole for the impedimetric sensing of AFP and CEA [25].

Amperometry monitors the electrical current generated during redox reactions, where electrons from indicators or products are transferred to electrode transducers after biochemical interactions. Measuring output current variation with applied fixed potential allows for highly sensitive analyte detection down to picomolar or femtomolar levels. For example, Fan et al. [20] developed a new amperometric immunosensor for detecting CEA using ZnMn_2_O_4_@reduced graphene oxide (ZnMn_2_O_4_@rGO) composites. The amperometric immunosensor was constructed by modifying a glassy carbon electrode with the ZnMn_2_O_4_@rGO composites and electrodepositing gold nanoparticles, which provided a substrate to immobilize CEA antibodies. The amperometric immunosensor leverages the high catalytic activity of ZnMn_2_O_4_@rGO composites towards hydrogen peroxide reduction to generate an amplified electrochemical signal (Figure 2A). When the CEA antigen binds to the anti-CEA antibodies immobilized on the ZnMn_2_O_4_@rGO modified electrode surface, it brings the ZnMn_2_O_4_@rGO nanocomposite into close proximity with the electrode. This facilitates more efficient electron transfer between ZnMn_2_O_4_@rGO and the electrode surface. Additionally, the binding event may cause a conformational change in the antibody that exposes more of the ZnMn_2_O_4_@rGO surface. The oriented alignment of ZnMn_2_O_4_@rGO through antibody binding provides easier access for hydrogen peroxide to reach the catalytically active sites on ZnMn_2_O_4_@rGO. This further promotes the electrocatalytic hydrogen peroxide reduction, leading to an enhanced amperometric current signal that is proportional to the concentration of CEA. The immunosensor has a broad linear detection range from 0.01 to 50 ng/mL and a low limit of detection of 1.93 pg/mL. Chronoamperometry determines current changes over time, enabling kinetic analysis [21]. Pulsed variants apply potential pulses for improved performance. Voltammetric techniques like cyclic voltammetry or differential pulse voltammetry (DPV) offer enhanced current analysis with changing potentials [22].

In potentiometry, equilibrium electrode potentials are recorded upon ionic interactions with selective membrane layers. Operation at zero current allows for obtaining measurements even in highly resistive samples. For example, Fu et al. [23] developed a new potentiometric immunoassay using magnetic nanorods for the detection of CEA in human serum samples. They synthesized uniform Fe_3_O_4_ nanorods with an average diameter of 40 nm and length of 1 μm. The nanorods were then functionalized with anti-CEA antibodies to create magnetic bio-nanorods. These antibody-conjugated nanorods were immobilized onto the surface of a carbon paste electrode using an external magnet. The binding of CEA antigens in serum samples to the anti-CEA antibodies changed the surface charge density, which was measured potentiometrically (Figure 2B). This label-free detection method had a dynamic range from 1.5 to 80 ng/mL CEA with a 0.9 ng/mL limit of detection. Hong et al. [24] also presented a new potentiometric aptasensor for detecting the cancer biomarker CEA. The aptasensor was constructed on a graphene oxide nanosheet-modified glassy carbon electrode (GCE). CEA aptamers were immobilized on the nanosheets via π-stacking interaction. Upon introduction of target CEA, the aptamer reacted with CEA, causing dissociation of the aptamer from the nanosheets. This triggered a measurable change in electrical potential due to the negatively charged DNA backbone. To amplify the signal, the authors coupled this system with DNase I-assisted target recycling. DNase I digested the CEA-aptamer complex, releasing CEA to react again with aptamers on the nanosheets. This resulted in cycling to detach numerous aptamers per CEA molecule, amplifying the potentiometric signal. The key performance achieved included a wide linear detection range of 0.01–100 ng/mL CEA and a low limit of detection of 9.4 pg/mL.

Impedimetric sensors rely on changes in ionic double-layer capacitance or electron transfer resistance arising from biomolecular binding events or architectural modifications. AC sinusoidal voltages are applied across wide frequency ranges to probe surface impedance changes. Conductive polymers or nanocomposites act as sensitive transducer materials. For example, Taheri et al. [25] developed a dual-template molecularly imprinted polymer (DMIP) electrochemical sensor using polypyrrole and methyl orange for the label-free impedimetric detection of the lung cancer biomarkers AFP and CEA (Figure 2C). The purpose was to create a sensor that could detect AFP and CEA sequentially in a simple, low-cost way without needing labels. They electrodeposited the DMIP sensing layer onto a fluorine-doped tin oxide electrode. The morphology was rectangular nanotubes which provided good conductivity and binding affinity. Using impedimetric detection, they measured changes in charge transfer resistance as AFP and CEA rebound to their specific imprinted sites. They obtained detection limits of 3.3 pg/mL for AFP and 1.6 pg/mL for CEA.

In addition to the discussed techniques, DPV and square wave voltammetry (SWV) are also commonly employed in electrochemical biomarker sensing. DPV applies a series of regular voltage pulses superimposed on a linearly increasing potential, measuring the current immediately before each potential change. The differential current peak height is directly proportional to the analyte concentration. SWV uses a symmetrical square-wave waveform superimposed on a staircase potential ramp. The current is sampled twice during each square-wave cycle, at the end of the forward and reverse pulses. The difference between these two currents is plotted versus the applied base potential. Both DPV and SWV provide excellent sensitivity by minimizing the contribution of the charging background current. The peak-shaped voltammograms also enable better peak resolution compared with conventional cyclic voltammetry. Several studies have demonstrated the use of DPV and SWV for the ultrasensitive quantification of lung cancer biomarkers like CEA, NSE, and AFP [26,27,28,29,30,31].

### 2.2. Electrochemical Immunosensors and Aptasensors

Immunosensors employ antibodies or antigens as biorecognition elements to quantitate respective analytes based on highly specific immunocomplex formation. Attached to transducer surfaces like gold electrodes, they detect biomarkers via changes in electrical properties after antibody–antigen binding. Using secondary detector antibodies enhances sensitivity through the amplification of output signals.

**Figure 3:**
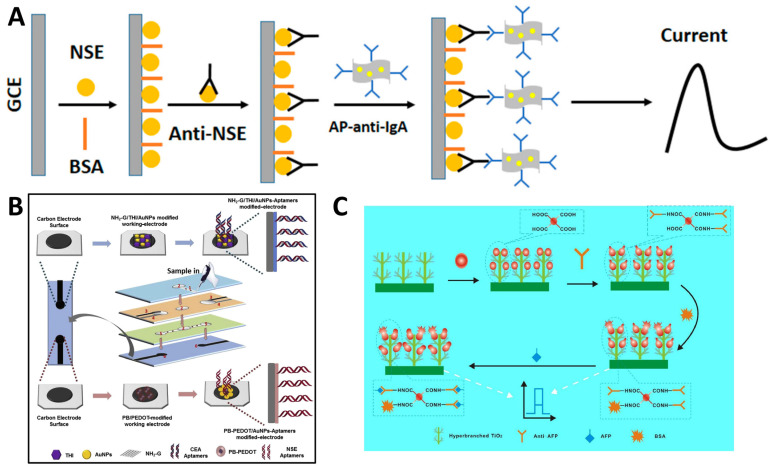
(**A**) An electrochemical immunosensor using a AuNP-RGO nanocomposite to detect NSE [32]. (**B**) Fabrication and modification process of the multi-parameter electrochemical paper-based aptasensor [33]. (**C**) TiO_2_-based PEC biosensor for detecting AFP [34].

For example, Wei et al. [32] developed an electrochemical immunosensor using a gold nanoparticle-reduced graphene oxide (AuNP-RGO) nanocomposite to detect NSE, a biomarker for lung cancer, with high sensitivity (Figure 3A). They employed an environmentally friendly method to synthesize the AuNP-RGO nanocomposite using chitosan as a green reducing and stabilizing agent. The numerous active sites on the AuNP-RGO nanostructure significantly enhanced the sensitivity of the immunosensor. Using alkaline phosphatase-conjugated antibodies to label the AuNP-RGO nanoprobe enabled dual signal amplification for detecting low NSE concentrations. The immunosensor exhibited a linear detection range from 0.1 ng/mL to 2000 ng/mL NSE with a limit of detection of 0.05 ng/mL.

Aptamers composed of single-stranded DNA/RNA oligonucleotides designed to bind unique target sites via shape-specific complementary base pairing and folding have emerged as robust biorecognition elements. Their stability, cost-effectiveness, and ease of chemical modifications make aptasensors popular for cancer diagnosis. For example, Wang et al. [33] presented a multi-parameter electrochemical paper-based aptasensor for the simultaneous detection of two cancer biomarkers, CEA and NSE, in clinical samples. The device was fabricated using wax printing and screen printing to create microfluidic channels and electrodes on paper. Two types of nanocomposites containing amino functional graphene, thionine, gold nanoparticles, Prussian blue, and poly(3,4-ethylenedioxythiophene) were synthesized and used to modify separate working electrodes. DNA aptamers with thiol groups were then immobilized onto the nanocomposites to recognize CEA and NSE specifically (Figure 3B). The aptamers provided high affinity and selectivity for the biomarkers. A label-free electrochemical detection method measured changes in peak currents to quantify the levels of antigens. The aptasensor showed good linearity from 0.01 to 500 ng/mL for CEA and 0.05 to 500 ng/mL for NSE, with limits of detection of 2 pg/mL and 10 pg/mL, respectively.

Photoelectrochemical (PEC) detection and electroluminescent labels are newer aptasensor trends that improve sensitivity. Aptamers directly grafted onto nanomaterial-modified electrode surfaces promote electron transfer too. With high specificity rivaling antibodies, tunable size, and high stability, aptasensors constitute promising candidates for early diagnosis of cancers via liquid biopsy. Polymeric substrates and nanomaterial augmentation enhance durability for cost-effective cancer screening applications. Mo et al. [34] developed a novel PEC immunosensor using a conjugated polymer-modified hyperbranched titanium dioxide platform to detect AFP (Figure 3C). The conjugated polymer with multi-carboxylic groups was synthesized and characterized. Modifying the TiO_2_ with the conjugated polymer shifted its light absorption from the ultraviolet to the visible range, reducing potential protein damage. The conjugated polymer also enhanced the photoelectric properties. The anti-AFP antibody was immobilized on the conjugated polymer–TiO_2_ surface. Under optimal conditions, the immunosensor exhibited a linear detection range of 0.1–100 ng/mL for AFP with a 0.03 ng/mL limit.

### 2.3. Signal Amplification Strategies

The inherently low abundance of cancer biomarkers poses sensitivity challenges for clinical diagnosis, demanding novel amplification methodologies to enhance output signals in electrochemical biosensors. Strategies encompass electron transfer mediation, high surface area nanomaterials, catalytic labels, and enzyme biocatalysis.

Nanomaterials like AuNPs, graphene, and carbon nanotubes (CNTs) facilitate sensor fabrication with high conductivity and surface area to allow for enhanced electroactive tagging for signal gains [35,36,37]. For example, Feng et al. [38] discussed recent advances in CNT-based electrochemical immunosensors for detecting protein cancer biomarkers. Their paper focused specifically on the use of CNTs, which have favorable conductivity, biocompatibility, and surface area properties, to improve electrode platforms and signal labels in these immunosensors. Two main strategies incorporating CNTs were outlined as follows: using CNTs or CNT hybrid materials to modify electrode platforms to achieve efficient electron transfer and increase antibody binding and using CNTs or functionalized CNTs as labels for secondary antibodies to enhance detection signals in sandwich-type immunosensors. Various CNT composites were summarized, including polymers, metals, ionic liquids, and mediators. Several recent immunosensors utilizing combinations of CNT platforms and labels to detect common cancer biomarkers at very low detection limits through signal amplification were highlighted.

**Figure 4:**
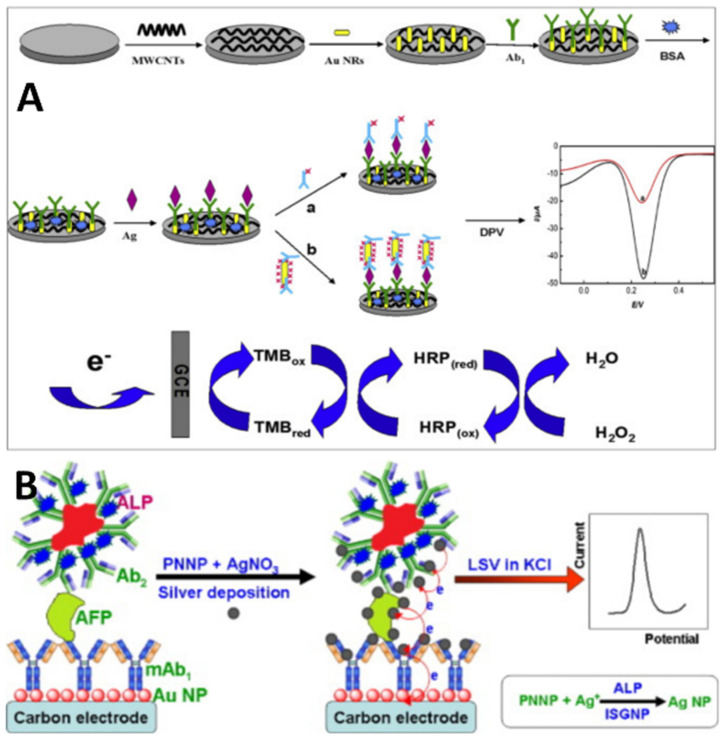
(**A**) HRP-Au NR bioconjugate for signal amplification of AFP [39]. (**B**) ISGNP-labeled ALP for AFP detection [40].

Enzymatic labels like horseradish peroxidase (HRP) or alkaline phosphatase (ALP) conjugate secondary antibodies to catalyze redox indicator substrates or precipitate insulating layers, respectively. For example, Guo et al. [39] developed a novel electrochemical immunosensor for detecting AFP. They employed a tracer made of HRP conjugated to gold nanorods (Au NRs) to label signal antibodies and paired this with a CNT/Au NR-modified electrode interface. The HRP-Au NR bioconjugate allowed for better signal amplification compared with labels without Au NRs (Figure 4A). The assay operated through a sandwich format that captured AFP between immobilized capture antibodies and the HRP-Au NR detection antibodies. Differential pulse voltammetry was then used to detect the response. The immunosensor provided a linear range from 0.1 to 100 ng/mL AFP and a limit of detection down to 30 pg/mL. Lai et al. [40] developed an electrochemical immunoassay for the detection of AFP using irregular-shaped gold nanoparticles (ISGNPs) labeled with ALP-conjugated antibodies. The purpose was to use the ISGNPs to increase enzyme loading and improve sensitivity compared with spherical AuNPs. The assay used a sandwich format on a carbon electrode modified with anti-AFP capture antibodies. After target binding and labeling with ALP-conjugated detection antibodies on ISGNPs, silver was deposited on the electrode through the catalytic activity of ALP. The deposited silver resulted in increased electrochemical signals upon voltammetry analysis that were proportional to AFP levels (Figure 4B). Compared with spherical gold nanoparticle labels, the ISGNP labels resulted in 5-fold increased signals because of higher enzyme loading. The immunoassay showed a broad detection range of 0.01–200 ng/mL AFP and a low detection limit of 5 pg/mL. Glucose oxidase (GOx) is another option.

**Figure 5:**
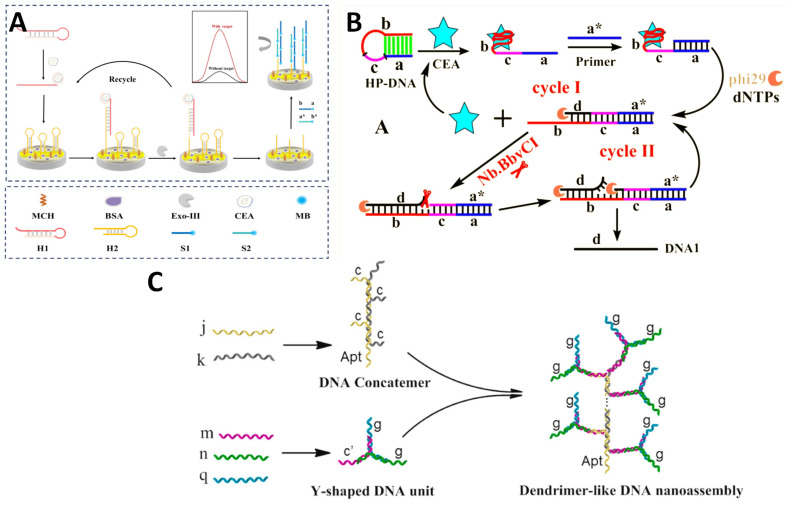
(**A**) Schematic illustration of an aptasensor for the detection of CEA based on Exo III and HCR dual signal amplification [41]. (**B**) ECL detection of CEA based on the target recycling amplification strategy [42]. (**C**) Preparation procedures for dendrimer-like DNA nanoassembly [43].

Nucleic acid amplification via polymerase chain reactions (HCRs), cycling probes, or DNA concatemers grow detectable oligonucleotide copy numbers for ultrasensitive gene biomarker quantification. Niu et al. [41] developed an electrochemical aptasensor for the detection of CEA. The aptasensor utilized exonuclease III (Exo III) and HCR for dual signal amplification. It worked by using an aptamer sequence to specifically recognize and bind CEA target molecules (Figure 5A). This triggered the assembly of complementary DNA hairpin structures on the sensor surface. Exo III then digested these structures, releasing new aptamers to bind more CEA molecules in a cyclic amplification. Meanwhile, HCR produced a long chain of methylene blue reporter molecules for further signal enhancement. This dual amplification strategy achieved a wide detection range of 10 pg/mL to 100 ng/mL and a low limit of detection of 0.84 pg/mL. Jie et al. [42] developed an ECL biosensor for the detection of CEA based on an amplification strategy. They prepared a novel Ru(bpy)_3_^2+^-doped SiO_2_ nanocomposite as the signal probe that has high ECL intensity. The signal probe was assembled on magnetic Fe_3_O_4_@Au nanoparticles. Then, a ferrocene-labeled DNA probe was introduced to quench the ECL signal (Figure 5B). In the presence of target CEA, a cyclic amplification process was triggered, producing a large amount of single-stranded DNA. These DNA strands displaced the ferrocene-labeled probes, restoring the ECL signal enhancement for the quantitative detection of CEA. Benefiting from the designed cascade signal amplification method, the developed ECL immunoassay achieved excellent performance with a wide detection range of 10 fg/mL to 10 ng/mL CEA and a low detection limit of 3.5 fg/mL. Zhai et al. [43] developed a label-free, antibody-free electrochemical aptasensor for detecting CEA. The biosensor utilized a dendrimer-like DNA nanoassembly made by the self-assembly of a DNA concatemer unit and a Y-shaped DNA unit. The DNA concatemer provided a high load of G-quadruplex DNAzyme motifs for signal amplification. A CEA aptamer was also incorporated to guide the binding of CEA to the sensor. The fabrication only required self-assembly of capture DNA on an electrode surface and hybridization to the CEA aptamer. Detection was based on CEA–aptamer binding releasing the aptamer and allowing the captured DNA to bind the DNA nanoassembly, bringing many G-quadruplex motifs to the sensor. This amplified the electrochemical signal for sensitive CEA quantification down to 0.24 ng/mL.

In addition to enhancing specific signal output, emerging signal amplification technologies also focus on minimizing non-specific background noise that can limit sensitivity. A key strategy is the use of antifouling surface chemistries that resist non-specific adsorption of interfering species. For example, coating electrode surfaces with zwitterionic polymers like poly(carboxybetaine methacrylate) or self-assembled monolayers terminated with oligoethylene glycol moieties has been shown to reduce background contributions from serum proteins or blood cells effectively [44]. The hydration layer formed around these coatings provides a physical and energetic barrier to non-specific binding. Another approach is the use of steric blocking agents like bovine serum albumin (BSA) or casein that passivate vacant surface sites after bioreceptor immobilization [45]. Novel nanocomposites that integrate specific biorecognition capability with antifouling properties offer a promising solution as well. Examples include the use of zwitterionic poly(sulfobetaine methacrylate) grafted onto reduced graphene oxide nanosheets [46] or the incorporation of PEGylated peptide aptamers into conducting polymer films [47]. Such materials simultaneously amplify target binding events while suppressing background noise. By integrating such technologies that address both signal enhancement and background suppression, electrochemical biosensors can attain exceptionally low limits of detection in complex clinical samples to enable early diagnostic applications against aggressive cancers like SCLC. While there is still progress to be made in extending the functional lifetime and storage stability of these antifouling materials, the field is rapidly advancing and holds great promise for the future development of ultrasensitive biosensing platforms.

## 3. Nanomaterials for Electrochemical Sensing

### 3.1. Carbon Nanomaterials

Owing to their high surface area, excellent conductivity, biocompatibility, and ease of functionalization, carbon nanomaterials like graphene and CNT have become indispensable for electrochemical cancer biomarker detection [48]. Graphene is a single-layer, two-dimensional lattice of sp^2^-hybridized carbon atoms [49]. Its large theoretical specific surface area of 2630 m^2^/g facilitates high-density loading of recognition elements like antibodies or aptamers for greater sensor sensitivity towards cancer biomarkers [50]. Additional merits are excellent mobility of charge carriers (2.5 × 10^5^ cm^2^/V·s) promoting rapid electron transfer and a high Young’s modulus (1.0 TPa) ensuring mechanical stability for sensor fabrication. Further, the presence of delocalized π-electrons enables easy grafting of functional groups via π–π stacking [51]. Common graphene variants employed are GO and rGO, which allow for straightforward bioconjugation chemistry for immobilizing bioreceptors [52]. Significant improvements in limiting detection ranges have been reported with graphene-based electrochemical immunosensors and aptasensors for cancer biomarkers.

**Figure 6:**
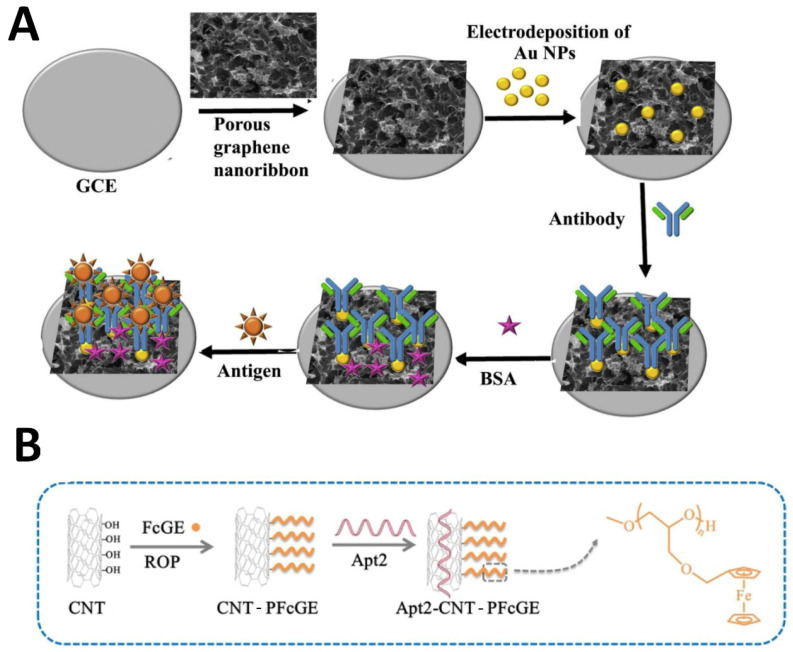
(**A**) AuNP/PGNR/GCE electrochemical immunosensor for the detection of AFP [53]. (**B**) Synthesis of Apt2-CNT-PFcGE via ROP [54].

For example, Jothi et al. [53] developed a novel label-free electrochemical immunosensor using a hybrid nanomaterial of AuNPs decorated on porous graphene nanoribbons (PGNRs) for the sensitive detection of AFP. PGNRs were prepared by chemically etching graphene oxide nanoribbons to create a porous structure, preventing restacking and increasing the surface area. AuNPs were then electrodeposited onto the PGNRs to provide a platform for antibody immobilization and facilitate electron transport. Anti-AFP antibodies were immobilized onto the AuNP/PGNR nanocomposite modified on a GCE (Figure 6A). In the presence of the AFP antigen, the antibodies specifically bound to AFP, hindering electron transfer and causing a detectable decrease in peak current that was proportional to the AFP concentration.

Similarly, CNTs prepared by folding graphene sheets comprise hollow graphitic cylinders with high tensile strength. Their availability as single-walled and multiwalled variants allows for extensive customization to obtain desired properties. High surface area (400–1000 m^2^/g), electric conductivity, and charge mobility aid superior electrocatalysis. Additionally, CNTs facilitate direct electron transfer with analytes. Polymer-functionalized CNTs composites can be used for the detection of CEA [54]. CNTs were grafted with abundant electroactive poly(ferrocenyl glycidyl ether) polymers via ring-opening polymerization (Figure 6B). This resulted in PFcGE-CNT composites that provided a conductive nanoplatform for signal amplification. The aptasensor was fabricated by immobilizing a CEA-binding aptamer probe on an electrode, followed by the capture of the CEA antigen and subsequent binding to a second CEA aptamer-labeled PFcGE-CNT composite. The ferrocene tags on the polymer side chains served as internal redox reporters. Testing showed a wide detection range from 1 fg/mL to 10 ng/mL CEA and a limit of detection down to 0.28 fg/mL.

### 3.2. Two-Dimensional Materials

In recent years, 2D nanomaterials beyond graphene have garnered significant interest for electrochemical biosensing applications because of their unique physicochemical properties. MXenes are an emerging class of 2D transition metal carbides, nitrides, and carbonitrides with excellent conductivity, hydrophilicity, and functionalization possibilities [55]. Their layered structure provides a high surface area for biorecognition element immobilization. Ti_3_C_2_ MXene nanosheets have been employed as transducer materials in sandwich-type immunoassays for the ultrasensitive detection of CEA [56]. The MXene interface exhibited enhanced electron transfer and anti-fouling properties. Combining MXene with Au-Cu alloy nanoparticles enabled dual signal amplification to achieve femtogram-level detection limits.

Transition metal dichalcogenides (TMDs) like MoS_2_ are another promising category of 2D nanomaterials composed of hexagonal layers of metal atoms sandwiched between two layers of chalcogen atoms [57]. The semiconducting nature, abundant active sites, and ease of exfoliation into thin nanosheets make TMDs attractive for electrochemical sensing [58]. MoS_2_ nanosheets integrated into conducting polymer nanocomposites have enabled the sensitive and selective detection of lung cancer biomarkers [59]. Layer-by-layer assembly of MoS_2_ with cationic polymers like PDDA on microfluidic chips has also been explored for enhanced AFP quantification [60]. MoS_2_ acts as an effective electron mediator while the polymer improves stability and capture antibody loading. Other 2D materials being investigated include black phosphorous, boron nitride, and transition metal oxides. Their atomically thin layered structure, tunable bandgap, and high carrier mobility are advantageous for electrochemical biosensors [61]. With further research into surface modification strategies and assay optimization, 2D material-based nanocomposites can offer a powerful toolkit for designing next-generation cancer diagnostic devices. Their seamless integration with microfluidics and flexible substrates can enable low-cost, miniaturized point-of-care sensors for early detection and better disease management.

### 3.3. Metal Nanoparticles

Owing to their high conductivity, catalytic properties, biocompatibility and ease of synthesis, metal nanoparticles like gold, silver, copper, and platinum are extremely popular in fabricating electrochemical biosensors [62,63]. Their high surface-to-volume ratio allows for enhanced loading of recognition elements like enzymes, antibodies, or aptamers for improved sensitivity [64]. Moreover, facile bioconjugation via interactions with thiols, amines, or other functional groups present on biological moieties is possible [65].

AuNPs amplify electron transfer when integrated into immunosensor platforms as they accelerate redox reactions and provide greater surface for antibody immobilization [66]. AuNPs in composites with graphene or carbon nanotubes also promote biomolecule adsorption [67]. Zhao et al. [68] developed an electrochemical immunosensor for the simultaneous detection of AFP and PSA. The immunosensor was fabricated by immobilizing antibodies for AFP and PSA onto a GCE modified with AuNPs/rGO. Secondary antibodies conjugated to the redox probes Azure A and Fc were attached to gold nanoparticle-coated silica nanoparticles. In the presence of AFP and PSA, the redox probe labeled secondary antibodies bound to the antigens via a sandwich immunoassay. Differential pulse voltammetry was used to measure the oxidation signals of the Azure A and Fc redox probes. Well-resolved peaks at −0.48 V for Azure A and +0.12 V for Fc were obtained. The immunosensor exhibited a linear range from 0.01 to 25 ng/mL for AFP and 0.012 to 25 ng/mL for PSA. The detection limits were 3.3 pg/mL for AFP and 4 pg/mL for PSA.

Additionally, metallic silver [69,70], copper [71,72], and nickel [73,74] nanostructures applied in cancer biosensing leverage properties like high catalytic activity and biocompatibility. Silica nanoparticles also assist high-density antibody loading and prevent leaching. Krishnan et al. [75] developed an electrochemical immunoassay for the detection of CEA using dual-labeled mesoporous silica nanospheres as a signal amplifier. The dual-labeled mesoporous silica (DLMS) nanospheres were synthesized by encapsulating gold nanorods and horseradish peroxidase enzyme within the channels of amine-functionalized mesoporous silica SBA-15, followed by conjugation of secondary antibodies onto the SBA-15 surface.

### 3.4. Conducting Polymers

Conducting polymers like polyaniline (PANI), polypyrrole (PPy), and polythiophene (PTh) have gained immense popularity in fabricating electrochemical biosensors owing to properties like biocompatibility, high conductivity, signal transducing ability, and easy synthesis [76]. Their versatile chemistry facilitates straightforward biofunctionalization [77]. Redox switching among different oxidation states leads to measurable electronic parameter changes [78].

For example, a label-free and reagentless electrochemical immunosensor was developed for the detection of CEA by electrodepositing PANI nanowires onto a porous poly(3,4-ethylenedioxythiophene) (PEDOT) and ionic liquid composite film on a GCE [79]. PANI was specifically utilized because of its inherent electroactivity, charge storage stability, and ease of electrodeposition without the need for a template. Antibodies against CEA were then immobilized on the PANI nanowires. The PEDOT composite film served as a conductive, porous substrate for growing a network of PANI nanowires to increase the electrode’s surface area for improved biomolecule immobilization. The binding of CEA onto the immobilized antibodies hindered the redox reaction of PANI, causing a decrease in peak current that was proportional to the CEA concentration. Song et al. [80] reported a flexible free-standing electrochemical biosensor for detecting CEA based on a PPy nanocomposite film electrode. The authors synthesized a PPy composite film with a sandwiched structure by combining PPy doped with pentaerythritol ethoxylate (PEE-PPy) and PPy doped with 2-naphthalene sulfonate (2-NS-PPy). AuNPs were electrodeposited onto the PPy film and connected to a CEA aptamer to construct the biosensor. Without needing an additional substrate or current collector, this aptamer-functionalized film electrode served as a stand-alone biosensor. Wang et al. [81] developed a chitosan/PTh/cadmium telluride (CS/PTh/CdTe) nanocomposite electrode for the electrochemical detection of CEA. They directly synthesized the CS/PTh/CdTe nanocomposites via a one-step method and connected them with CEA antibodies. Using this electrode, they achieved an ultra-wide CEA detection range from 0.0001 to 10,000 ng/mL with a low detection limit down to 40 fg/mL. PTh was used because its good optoelectrical properties, biocompatibility, and ease of fabrication improved dispersion provided more antibody attachment sites and enhanced conductivity and the electron transfer rate.

## 4. Recent Advances in the Electrochemical Sensing of SCLC Biomarkers

Electrochemical biosensors have enabled the ultrasensitive detection of SCLC biomarkers from easily accessible biofluids like serum, urine, and saliva. Recent advances utilize nanomaterials like graphene and conducting polymers to enhance specificity and lower detection limits.

### 4.1. CEA Sensors

CEA is an extensively studied SCLC biomarker protein measured preoperatively for staging and postoperatively for recurrence monitoring [82]. Conventional CEA detection techniques like ELISA offer limited sensitivity around 2–5 ng/mL [83]. Electrochemical immunosensors and aptasensors now lower this limit by 1000-fold in some cases for early diagnosis.

Graphene-based metal nanocomposites are widely used owing to excellent conductivity and high surface area. Recently, Yan et al. [84] developed a CEA electrochemical aptasensor based on graphene nanosheets decorated with Ag NPs (GNSs@Ag NPs). The aptasensor was fabricated by modifying a screen-printed carbon electrode with the GNSs@Ag NPs composite and immobilizing a thiol-terminated CEA aptamer on its surface. The wide linear range was from 0.001 pg/mL to 10 pg/mL with a correlation coefficient of 0.9958. The achieved detection limit was 0.5 fg/mL based on the S/N = 3 protocol. The aptasensor demonstrated good selectivity towards CEA over potential interfering proteins. It also showed acceptable reproducibility with a relative standard deviation of 3.3% along with fast response time. The applicability of the developed strategy was examined in spiked human serum samples and compared to results from a commercial enzyme-linked immunosorbent assay, showing good accuracy.

Conducting polymer interfaces also enhance CEA recognition remarkably. PANI nanofibers electrodeposited on gold electrodes allowed for the immobilization of anti-CEA for sensitive impedimetric measurements down to 0.7 pg/mL and dynamic ranges up to 10 ng/mL CEA [79]. Recently, Feng et al. [85] developed an immunosensor for detecting CEA using AuNP-dotted Prussian blue@PANI (Au NPs/PB@PANI) core–shell nanocubes as a signal probe. They first synthesized uniform PB@PANI core–shell nanocubes and then attached gold nanoparticles to the surface to form the Au NPs/PB@PANI nanocomposite. They constructed a sandwich-type immunosensor by using these nanocubes along with gold–platinum bimetallic nanoflowers (AuPt NFs) electrodeposited onto a GCE modified with L-cysteine. This sensor detected CEA with a wide linear range from 1.0 pg/mL to 100 ng/mL and a low detection limit of 0.35 pg/mL. Table 1 summarizes the recently developed electrochemical CEA sensors with their performances.

### 4.2. NSE Sensors

As a cytoplasmic glycolytic pathway marker elevating in SCLC malignancies, sensitive quantification of NSE levels aids staging and prognosis appraisal [96]. An ultrasensitive immunosensor fabricated with MnO_2_ nanosheets labeling secondary antibodies and gold/palladium/platinum core–shell nanoparticles decorating a nanoflower platinum electrode reached a 4.17 fg/mL detection limit from 10 fg/mL to 100 ng/mL [97]. Among label-free immunosensors, a zirconium–porphyrin nanostructured film yielded a 7.1 fg/mL detection limit from 10 fg/mL to 2 ng/mL NSE [98]. Key challenges include properly orienting antibodies for maximum antigen binding, minimizing non-specific binding, reducing assay time, eliminating signal labels, and integrating nanomaterial-based electrochemical immunosensors into point-of-care devices. While significant advances have been made in lowering detection limits, accelerating assay times, and demonstrating applicability in clinical samples, more research is needed to transition highly sensitive laboratory-based NSE immunosensing tools into commercial point-of-care devices for early SCLC diagnosis.

Recently, a self-supported PtPdMnCoFe high-entropy alloy with nanochain-like internetworks (HEAINNs) was used for NSE detection [99]. The high-entropy alloy demonstrated excellent electrocatalytic efficiency towards H_2_O_2_ reduction because of its high surface area and synergetic “cocktail effects” of the multi-metals. The immunosensor achieved a broad linear detection range of 0.1 pg/mL to 200 ng/mL and an ultra-low limit of detection of 0.0036 pg/mL. Aydın et al. [100] developed a novel electrochemical immunosensor using an epoxy-substituted polypyrrole (P(Pyr-Epx)) polymer-modified disposable indium tin oxide (ITO) electrode for the detection of NSE. The P(Pyr-Epx) polymer provided an effective platform to immobilize anti-NSE antibodies and increased the electrode surface area. Under optimized conditions, the immunosensor showed a wide linear detection range from 0.02 pg/mL to 7.5 pg/mL for NSE with a low limit of detection of 6.1 fg/mL. Table 2 summarizes the recently developed electrochemical NSE sensors with their performances.

### 4.3. AFP Sensors

Though the literature on the electrochemical detection of glycoprotein biomarker AFP in SCLC is sparse, some key studies demonstrate ultrasensitive measurements in hepatocellular carcinoma applications employing electrodes engineered with graphene, conductive polymers, and nanocomposites. ELISA offers only modest sensitivity around 2–5 ng/mL for AFP, unlike advanced electrochemical aptasensors and immunosensors. Dual-probe electrochemical sensors using MB and ferrocene as signaling molecules provide sensitive detection similar to that of ECL sensors [109]. PEC sensors using quantum dots for surface modification have been commonly studied but do not achieve detection limits as low as other methods. For electrochemical sensors, modified electrodes with nanomaterials like AuNPs [110], CNT [111], and HRP [112] enhance signaling and catalytic activity. Monoclonal antibodies provide specific antigen recognition over polyclonal antibodies [113]. Dual-probe electrochemical sensors enhance signals in one direction while attenuating in the other, allowing for sensitive detection down to the ag/mL level. For PEC sensors, combinations of ZnO [114] and AuNPs as composites improve conductivity and photocurrent responses. ECL sensors frequently use quantum dots for modification and achieve the most sensitive detection limits. EC and ECL immunosensors demonstrate great capability for sensitive and selective AFP detection necessary for clinical use. Further innovations in dual-signaling molecules, quantum dots, and RNA binding probes can provide platforms for ultrasensitive and early detection of cancer biomarkers like AFP. Table 3 summarizes the recently developed electrochemical AFP sensors with their performances.

## 5. Biological Samples and Sensing

Electrochemical biosensors offer simple, rapid tools for the sensitive detection of SCLC biomarkers from diverse biological samples. However, matrix effects can impact their performance, necessitating optimization. As blood constituents mirror physiological status, serum is widely tested for circulating cancer biomarkers [120]. Mitigating interfering species like lipids, metabolites, and high-abundance proteins in serum is vital for analytical reliability. Strategies include physical separation via selective membranes, chemical modification of sensing interfaces, and the use of secondary labels.

**Figure 7:**
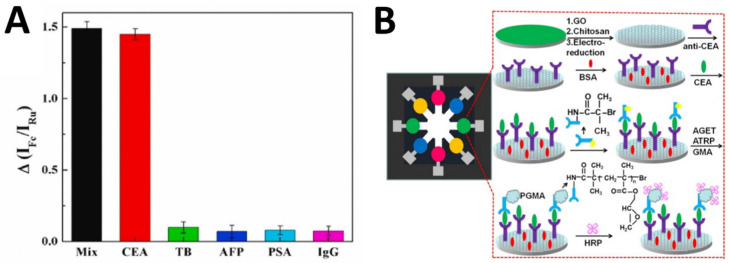
(**A**) The selectivity of MXC-Fe_3_O_4_-Ru to CEA [87]. (**B**) Schematic representation of a paper-based microfluidic electrochemical immunodevice for CEA sensing [126].

For example, one biosensor detects CEA in serum and avoids interference through the use of an antifouling nanocomposite interface called MXC-Fe_3_O_4_-Ru on a magnetic gold electrode (Figure 7A) [87]. On this interface, a CEA aptamer strand tagged with a ferrocene reporter is hybridized to capture CEA from serum samples. The binding of CEA causes detachment of the aptamer and a decrease in the ferrocene signal. Simultaneously, an internal standard ruthenium complex on the interface provides a constant reference signal. Taking the ratio of the reporter to internal standard signals enables accurate CEA quantification even in complex sera, as the MXC-Fe_3_O_4_-Ru composite resists non-specific fouling. Another work [23] describes a potentiometric immunoassay using magnetic iron oxide nanorods modified with anti-CEA antibodies. The nanorods are immobilized on a carbon paste electrode using an external magnet. The binding of CEA from the serum sample to the antibodies causes a change in surface charge that is detected as a change in potential. To avoid interference, optimal conditions such as pH, incubation time, and temperature are determined. The selectivity is also tested by adding potential interfering substances like AFP, CA 19-9, CA 125, and BSA at different concentrations.

Plasma resembles extracellular fluid, allowing for easier assessment of circulating biomarkers unlike intracellular contents found in serum [121]. A key concern, however, is plasma coagulation causing thrombotic depositions and blocking electrode surfaces. It is well established that protein adsorption onto electrode surfaces can hinder electron transfer and decrease sensor sensitivity. Noble metals like gold and platinum have been shown to resist non-specific protein binding because of their relatively inert and hydrophobic nature compared with other electrode materials. The adsorption of proteins onto gold and platinum surfaces has been extensively studied using various surface characterization techniques like quartz crystal microbalance (QCM), surface plasmon resonance (SPR), and atomic force microscopy (AFM). For example, Recek et al. [122] used QCM to compare the adsorption of BSA onto gold, platinum, and titanium oxide surfaces. They found that BSA adsorption was significantly lower on gold and platinum compared with titanium oxide. Furthermore, strategies to modify gold and platinum electrodes with self-assembled monolayers or polymers containing ethylene glycol or zwitterionic groups have been shown to further reduce protein fouling [123,124]. These antifouling coatings create a hydration layer that acts as a physical and energetic barrier to protein adsorption. To detect CEA in plasma, Ag@C nanocomposites were first modified onto a GCE [125]. Anti-CEA antibodies were then immobilized onto the Ag@C nanocomposites to capture CEA. The immunosensor worked by measuring changes in electrical current signal upon binding of CEA antigens to the anti-CEA antibodies. As more CEA bound to the antibodies, more insulation of the electrode surface occurred, decreasing the electrical current measured. So, by measuring decreases in current, the CEA level could be quantitatively detected through the established calibration curve. To avoid interference, the selectivity of the immunosensor was tested by detecting the current response in solutions with additional proteins that could potentially interfere, including BSA, AFP, IgG, and prostate-specific antigen (PSA). It was found that at the same concentrations, these proteins did not cause a significant change in the current response for CEA detection, indicating good selectivity of the immunosensor.

Microfluidic paper-based origami devices are emerging alternatives that allow for rapid wicking and preconcentration of dilute cancer biomarkers in small volumes onto sensor detection zones. For example, Wu et al. [126] described a novel signal amplification strategy using a paper-based microfluidic electrochemical immunodevice for the ultrasensitive detection of multiple cancer biomarkers. The device was fabricated by screen-printing electrodes onto patterned filter paper (Figure 7B). Activator-generated electron transfer atom transfer radical polymerization (AGET ATRP) was utilized to graft glycidyl methacrylate (GMA) polymers containing epoxy groups from the surface of the immunosensor after the capture of the target biomarkers. The polymer provided numerous sites for the immobilization of HRP enzyme labels, significantly enhancing the electrochemical signal. Detection was performed by chronoamperometry using the HRP-catalyzed hydrolysis of hydrogen peroxide. The signal was further amplified by modifying the immunosensor surface with graphene nanosheets to accelerate electron transfer. The device was used to simultaneously detect four cancer biomarkers—CEA, AFP, CA125, and CA153—with a short analysis time. The amplification strategy allowed for detection limits down to 0.01 ng/mL for CEA and AFP and 0.05 ng/mL for CA125 and CA153, which are clinically relevant concentration ranges. The disposable microfluidic immunodevice provided ultrasensitive detection combined with low cost, simplicity, and high throughput capabilities. Cross-reactivity studies showed negligible interference among working electrodes, enabling multiplexed detection.

## 6. Sensor Integration and Multiplexed Detection

Transferring electrochemical biosensors from lab settings into practical point-of-care devices remains challenging despite extensive research. This requires integration innovations across nanotechnology, microfluidics, and electronics for reproducible multiplexed detection from minute samples.

**Figure 8:**
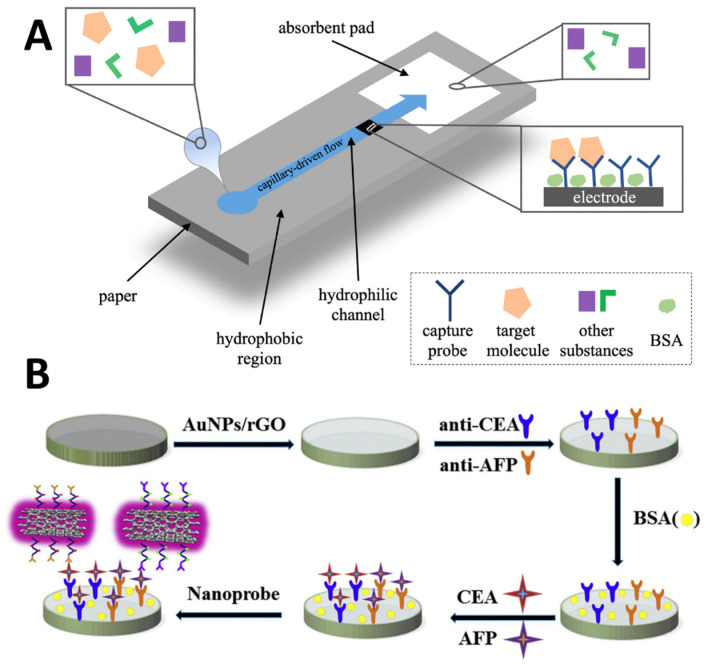
(**A**) The operational principle of electrochemical μPADs [127]. (**B**) AN electrochemical immunosensor for the simultaneous detection of CEA and AFP [132].

Leveraging microelectromechanical advances, miniaturized point-of-care biochips enable rapid measurements of cancer biomarker panels at a fraction of central laboratory testing costs. For example, Yuan et al. [127] reported using electrochemical microfluidic paper-based analytical devices (μPADs) to detect common tumor markers for cancer screening and diagnosis (Figure 8A). μPADs are miniaturized paper platforms that allow fluid manipulation without external pumps. By integrating electrochemical detection methods like voltammetry, potentiometry, amperometry, and impedance spectroscopy, μPADs enabled a rapid, low-cost, and sensitive analysis of cancer biomarkers. They first discussed fabrication techniques to create hydrophobic barriers on paper to form microfluidic channels. Methods like wax printing, inkjet printing, screen printing, laser treatment, and stamping have been reported. Strategies to construct paper-based 3D devices have also been summarized, which introduce vertical flow control and minimize sample volume compared with 2D lateral devices. The integration of screen-printed or inkjet-printed electrodes turns paper substrates into low-cost electrochemical sensors. Various cancer protein biomarkers like PSA [128], CEA [129], AFP [130], and cytokines [131] have been detected by μPADs down to picogram per milliliter levels. By functionalizing electrodes with nanomaterials and redox reporters, both label-free and signal-amplified strategies have been applied. Nucleic acids associated with cancers such as miRNA and DNA have also been successfully analyzed by μPADs using voltammetry or impedimetry. Despite the significant progress, limitations exist including the variability in paper-based devices, sample evaporation, and stability issues. The sensitivity for early cancer detection still needs to be improved. Future efforts are expected in 3D construction and multichannel integration as well as applying artificial intelligence for data analysis.

Detecting several complementary cancer biomarkers offers superior diagnostic reliability compensating for the inadequacies of single markers that show poor specificity. Electrochemical immunosensor arrays thus aim to measure panels of antigens, enzymes, mutated genes, or glycoproteins simultaneously from minute samples. However, this demands avoiding sensor crosstalk. For example, Wang et al. [132] developed an electrochemical immunosensor using MWCNTs modified with poly(glycidyl methacrylate) polymer brushes (PGMA) for the simultaneous detection of CEA and AFP (Figure 8B). The PGMA brushes were grown from the MWCNT surface using surface-initiated photoinduced electron transfer–atom transfer radical polymerization with a mussel-inspired polydopamine coating. The epoxy groups of the PGMA were then reacted with ethylenediamine to provide binding sites for signal molecules anthraquinone-2-carboxylic acid and ferrocenecarboxylic acid as well as capture antibodies. The electrochemical immunosensor, using a glassy carbon electrode modified with rGO and AuNPs, achieved the simultaneous detection of CEA and AFP over wide linear ranges down to low detection limits of 56.1 fg/mL for CEA and 32.8 fg/mL for AFP. Yang et al. [133] developed an electrochemical immunosensor for the simultaneous detection of two lung cancer biomarkers, cytokeratin 19 fragment antigen 21-1 (CYFRA21-1) and CEA. The immunosensor was fabricated by modifying a glassy carbon electrode with three-dimensional graphene (3D-G) and AuNPs to provide a large surface area for antibody binding. Two polymer nanocomposites, poly-thionine-AuNPs (Au-pThi) and poly-m-cresol purple-AuNPs (Au-pMCP), were synthesized and covalently conjugated to antibodies for CYFRA21-1 and CEA to act as signal tags. Au-pThi and Au-pMCP showed enhanced electrochemical signals compared with the unmodified polymers, allowing for more sensitive detection. The immunosensor exhibited linear detection ranges of 0.5–200 ng/mL for both CYFRA21-1 and CEA with low limits of detection of 0.18 and 0.31 ng/mL, respectively.

Microfluidic integration offers automated, rapid analysis from microliter patient volumes with scope for high-throughput screening. Valve and pump integration permits precise sample handling while surface modification allows for biomarker capture across reacted zones instead of localized spots. Dhanapala et al. [134] explained that SPEs and inkjet-printed electrodes provide low-cost, reproducible platforms that can be easily integrated with microfluidics for developing point-of-care diagnostic devices. They discussed common immunoassay protocols on printed electrodes, including layer-by-layer antibody attachment, strategies for signal amplification using nanomaterials and multi-enzyme labels, and label-free detection methods. They highlighted work from their group and others showing that combining nanostructured metal nanoparticles, carbon nanomaterials, and/or magnetic microbeads decorated with thousands of enzyme labels enables ultrasensitive detection down to femtogram per mL levels for cancer biomarkers. For example, their four-protein multiplexed immunoarray using SPEs modified with glutathione-capped AuNPs and secondary antibodies labeled with streptavidin-polyHRP achieved sub-zeptomole detection limits [135]. They also summarized key fabrication details and advantages of screen-printing and inkjet-printing technologies. For example, Hu et al. [60] developed an electrochemical biosensor based on a microfluidic chip with a working electrode made of self-assembled MoS_2_/PDDA hybrid films. The biosensor was designed to detect AFP. Using a layer-by-layer approach, they deposited ten layers of alternating MoS_2_ nanosheets and positively charged PDDA to form a porous film with a high surface area on an indium tin oxide electrode. After activating the carboxyl groups on the hybrid film, they immobilized anti-AFP antibodies to enable the selective binding of AFP antigens. Electrochemical impedance spectroscopy was used to detect changes in the specific binding of AFP antigens to the antibodies over a range of AFP concentrations. A linear detection range from 0.1 ng/mL to 10 ng/mL was demonstrated, with an extremely low limit of detection reaching 0.033 ng/mL. The microfluidic design enabled low sample consumption.

In summary, while individual electrochemical sensors already offer impressive performance as standalone units, their clinical translation relies on seamlessly incorporating sample processing, calibration protocols, validation filters, and connectivity while retaining ease of use and affordability. This necessitates interdisciplinary efforts across biomedical, mechanical, and computer engineering for developing next-generation smart integrated biosensing devices for early cancer screening.

## 7. Challenges and Future Outlook

Despite the tremendous progress made in electrochemical biosensing, several challenges need to be addressed for widespread clinical adoption and patient benefit. While many novel nanomaterial-based electrochemical biosensors show impressive sensitivity and specificity for SCLC biomarkers in controlled lab settings, translating this performance to real-world clinical applications can be hindered by issues like batch-to-batch variability in nanomaterial synthesis, surface functionalization, and bioconjugation. Subtle differences in these parameters can significantly impact sensor reproducibility. There is a pressing need for more rigorous optimization and standardization of these fabrication processes to ensure consistent sensor performance across different production batches and testing sites. This includes establishing standard operating procedures and quality control metrics and possibly exploring automated and scalable manufacturing approaches. Furthermore, there is currently a paucity of large-scale, multi-site clinical validation studies for these electrochemical biosensors. Conducting such trials is crucial for establishing robust performance in real patient samples across diverse populations. Factors like sensor stability during storage, matrix effects of clinical samples, and establishing universal cutoff/threshold values need to be thoroughly evaluated through large patient cohort studies. Incorporating sample pre-treatment steps, blocking reagents against nonspecific binding, and multi-biomarker panel detection can help mitigate potential variability. Greater standardization and compliance with clinical Good Manufacturing Practices in component sourcing and scale-up synthesis of functional nanomaterials used in these biosensors is also essential. Variations in raw materials and synthesis parameters can lead to inconsistent sensor performance. Establishing standard sources, synthesis protocols, and characterization criteria (particle size, surface area, functionalization density, etc.) will be key for future clinical translation and commercial viability.

Closer collaboration among different stakeholders can shape future trajectories beneficially. Firstly, device engineering aspects like microfluidic integration, electronics interfacing for signal processing, and portable packaging need further innovation from a stability and reproducibility perspective before health certification for productization. Component sourcing and scale-up synthesis require standardization for batch-to-batch consistency. Sensor surface chemistries need optimization against interfering species in the intended sample matrix through rigorous testing across heterogeneous patient cohorts spanning various demographics and disease stages. As sensors inch closer to clinics eventually, pragmatic factors like training technicians, reimbursement policies, and electronic health record integration need consideration well in advance.

Another key facet is the need for exhaustive multi-site validation studies not just confirming technical viability but also carefully evaluating feasibility for supporting and improving therapeutic decisions clinically via earlier diagnosis or optimal interventions against aggressive small cell lung cancer. Comparing diagnostic yields against conventional biopsy and radiology approaches using robust statistical tests for sufficient analytical evidence is vital. Demonstrating additive value either through delineating prognostic subgroups or by complementing histopathology information with molecular profiling is desired, as that can accelerate adoption and acceptance amongst practicing oncologists, clinicians, and patients alike.

Going forward, closer collaboration among material scientists, engineers, and clinicians is indispensable to steer biosensor innovation towards implementations that best address unmet needs in combating lung cancer effectively by early definitive screening using liquid biopsy. Advances in synthetic biology also hold promise for the engineering and use of radically novel alternative binders beyond conventional antibodies with higher stability or entirely new recognition modalities. Wider accessibility facilitated by connectivity with smartphone healthcare apps allows for decentralized testing, alleviating the need for specialized infrastructure as well. Periodic technology assessment reviews can guide supportive policy aimed at faster translational success and public health benefits.

IoT connectivity could enable real-time data transmission from these point-of-care devices to centralized databases, allowing for continuous remote monitoring of patient health status. This could facilitate timely clinical interventions and personalized treatment decisions. To realize this vision, biosensors would need to be interfaced with wireless communication modules and microcontrollers to enable secure data transfer. Cloud computing platforms could be leveraged for data storage, analysis, and sharing among authorized healthcare providers. Developing user-friendly mobile applications that display actionable insights derived from biosensor data would also be crucial for patient engagement and compliance. Moreover, integrating machine learning algorithms could further enhance diagnostic performance by identifying complex biomarker patterns and predicting disease progression. However, robust data encryption and privacy protection measures would be essential to ensure patient confidentiality. While there are technical challenges to overcome, such as ensuring sensor stability and data reliability in remote settings, the integration of IoT capabilities with electrochemical biosensors holds immense potential for revolutionizing cancer management through decentralized, data-driven approaches.

The clinical adoption of advanced electrochemical biosensing technologies for SCLC biomarkers raises important ethical and regulatory considerations that need to be carefully addressed. While these ultrasensitive biosensors offer the potential for early detection and improved patient outcomes, their implementation in clinical settings may have unintended consequences. The major ethical concern is the potential for overdiagnosis and overtreatment. The ability to detect extremely low levels of SCLC biomarkers may lead to the identification of indolent or slow-growing tumors that may not have clinical significance. This could subject patients to unnecessary invasive diagnostic procedures and treatments, leading to increased healthcare costs and psychological distress. To mitigate this, it is crucial to establish appropriate clinical decision-making protocols and risk–benefit assessment frameworks that guide the interpretation and actionability of biosensor results. This may involve setting evidence-based threshold values, incorporating patient preferences and quality of life considerations, and developing personalized risk stratification algorithms. From a regulatory perspective, obtaining approval for these novel biosensing devices presents its own set of challenges. Demonstrating robust analytical and clinical validity, as well as cost-effectiveness compared with existing diagnostic methods, will be key for regulatory clearance. This will require close collaboration among researchers, device manufacturers, and regulatory bodies to establish standardized performance evaluation criteria, quality control guidelines, and post-market surveillance protocols specific to these advanced electrochemical biosensors. Navigating the evolving regulatory landscape and ensuring compliance with applicable directives and standards will be an ongoing process.

## 8. Conclusions

Nanocomposite-based electrochemical biosensors are revolutionizing lung cancer diagnosis by enabling non-invasive, rapid, and ultrasensitive quantification of protein and genetic biomarkers like CEA, NSE, and AFP from liquid biopsies. Nanomaterials like graphene, metal nanoparticles, and conducting polymers have played a pivotal role in lowering detection limits from clinically relevant fg/mL to pg/mL levels by enhancing electron transfer, biocompatibility, and bioreceptor loading. However, unleashing the full potential of these sensors demands rigorous optimization of parameters like nanomaterial synthesis, surface chemistry, assay design, and antifouling properties to ensure reliable performance in complex clinical samples. Establishing standardized protocols for quality control and validation will be critical for mitigating technical variability. From a practical standpoint, greater efforts in system integration, multiplex biomarker panels, and clinical feasibility studies are vital to motivate real-world adoption. Ultimately, the path to saving lives lost to lung cancer will be paved by strategic and sustained collaboration across the scientific community. Material scientists need to work closely with clinical chemists to engineer robust sensor interfaces. Oncologists and pathologists need to identify the most informative biomarker signatures. Engineers need to transform proof-of-concept devices into user-friendly, automated sample-to-answer systems. Regulatory agencies need to define clear approval pathways based on measurable patient outcomes. The future is primed for innovation in smart biosensors that can decode molecular signatures of lung cancer from a simple blood draw to inform clinical decisions. Electrochemical biosensors enhanced with nanocomposites are poised to be at the forefront of this revolution, empowering personalized early interventions to turn the tide against this deadly malignancy.

## Figures and Tables

**Table 1:** Recently developed electrochemical CEA sensors with their performances.

Sensing Strategy	Technology	Linear Detection Range	Limit of Detection	Real Sample	Ref.
Self-assembled, label-free 3D DNA nanoprobe and exonuclease III-assisted signal amplification	DPV	10 fg/mL to 50 ng/mL	4.88 fg/mL	Serum	[86]
Ratiometric electrochemical detection using an aptamer and an internal standard	DPV	1 pg/mL to 1 μg/mL	0.62 pg/mL	Serum	[87]
Sandwich-type assay using primary anti-CEA antibody immobilized on MWCNT/CuO nanocomposite-modified electrode, CEA antigen, and secondary anti-CEA antibody conjugated to Fe_3_O_4_ nanoparticles	DPV	0.005 ng/mL to 4 ng/mL	1.9 pg/mL	Serum	[88]
Sensing strategy exonuclease III and hybrid chain reaction dual signal amplification	I-T	10 pg/mL to 100 ng/mL	0.84 pg/mL	Serum	[41]
Label-free, electric field-mediated electrochemical detection using a graphene–ZnO nanorod heterostructure	EIS	0.001 pg/mL to 10 pg/mL	1 fg/mL	-	[89]
Electrochemical aptamer biosensor based on tetrahedral DNA nanostructures and catalytic hairpin assembly	DPV	1 pg/mL to 30,000 pg/mL	0.04567 pg/mL	Serum	[90]
Electrochemical immunosensor based on redox probe-modified electron transfer wires and an immobilized antibody	DPV	10 pg/mL to 100 ng/mL	0.6 pg/mL	Serum	[91]
Electrochemical immunosensor with RCA	DPV	0.01 pg/mL to 80 ng/mL	0.0037 pg/mL	Serum	[92]
Sandwich-type electrochemical immunosensor using magnetic hollow Ni/C@SiO2 nanomatrix and a boronic acid-functionalized CPS@PANI@Au probe	DPV	0.006–12.00 ng/mL	1.56 pg/mL	Serum	[73]
Electrochemical immunosensor using Ti_3_C_2_ MXene-anchored CuAu-LDH as signal enhancer	I-T/DPV	0.0001–80 ng/mL	33.6 fg/mL	Serum	[93]
Enzyme-free sandwich-type electrochemical immunosensor using a Ag/g-C_3_N_4_-modified electrode and a Au@SiO_2_/Cu_2_O signal probe	I-T	0.01 pg/mL to 80 ng/mL	0.0038 pg/mL	Serum	[94]
Label-free electrochemical immunosensor based on graphene oxide	EIS	0.1 to 5 ng/mL	0.05 ng/mL	Serum	[95]

**Table 2:** Recently developed electrochemical NSE sensors with their performances.

Sensing Strategy	Technology	Linear Detection Range	Limit of Detection	Real Sample	Ref.
Label-free electrochemical immunosensor using PtPdMnCoFe HEAINN as signal amplifier	DPV	0.1 pg/mL to 200 ng/mL	0.0036 pg/mL	Serum	[99]
Label-free electrochemical impedimetric immunosensor using an epoxy-substituted polypyrrole (P(Pyr-Epx)) polymer-modified disposable ITO electrode	EIS	0.02 pg/mL to 7.5 pg/mL	6.1 fg/mL	Serum	[100]
Label-free electrochemical immunoassay based on anti-NSE antibodies immobilized on a AuNP-modified conductive hydrogel film	DPV	1 pg/mL to 200 ng/mL	0.26 pg/mL	-	[98]
Sandwich-type electrochemical immunosensor using Au/Cu x O@CeO_2_ as label material and AuPt NSNs as substrate	I-T	50 fg/mL to 100 ng/mL	31.3 fg/mL	Serum	[101]
Sandwich immunoassay using anti-NSE21 antibody modified with disulfide groups via carbohydrate residues as the capture antibody and anti-NSE17-HRP conjugate as the reporter antibody	DPV	0–25 ng/mL	4.6 ng/mL	-	[102]
A 3D graphene–starch-modified immunoelectrode to capture antigens, AuNP-loaded antibody tags to catalyze silver deposition, and direct detection of AgNPs using stripping voltammetry for signal amplification	LSV	0.02 pg/mL to 35 ng/mL	0.008 pg/mL	Serum	[103]
PtCu nanoprobe-initiated cascade reaction and iodide-responsive sensing interface	SWV	0.0001 to 100 ng/mL	52.14 fg/mL	Serum	[104]
Sandwich-type electrochemical immunosensor using HP-AgPt/NGR as a dual signal amplification label and PPy-PEDOT-Au as the substrate	I-T	50 fg/mL to 100 ng/mL	18.5 fg/mL	Serum	[105]
Ratiometric electrochemical immunosensor based on Cu-MOF-Au as the electrode sensing surface and Fc-L-Cys as the label of Ab2	DPV	1 pg/mL to 1 μg/mL	0.011 pg/mL	Serum	[106]
An electrochemical NSE immunosensor using a AuNPs@MoS_2_/rGO platform and a CoFe_2_O_4_@Ag label for signal amplification	DPV	0.01 to 1.00 pg/mL	3.00 fg/mL	Serum	[107]
PEC immunosensing using ZnO/CdSe and an antifouling interface	DPV	0.10 pg/mL–100 ng/mL	34 fg/mL	Serum	[108]

**Table 3:** Recently developed electrochemical AFP sensors with their performances.

Sensing Strategy	Technology	Linear Detection Range	Limit of Detection	Real Sample	Ref.
Electrochemical immunosensor based on a AuNP–dextran–rGO nanocomposite	DPV	0.01–20 ng/mL	0.05 pg/mL	Serum	[110]
Electrochemical immunosensing using an anti-alpha fetoprotein antibody labeled with horseradish peroxidase immobilized on poly-L-lysine-functionalized SWCNT/PB composite film	DPV	0.05–10.0 ng/mL 10.0–50.0 ng/mL	0.011 ng/mL	Serum	[111]
Chemiluminescent immunoassay based on dual signal amplification using HRP and an HRP-labeled antibody co-immobilized on mesoporous silica nanoparticles	ECL	0.01 to 0.5 ng/mL 0.5 to 100 ng/mL	0.005 ng/mL	Serum	[112]
Sandwich-type electrochemical immunosensor using a signal amplification strategy	DPV	0.02–10,000 pg/mL 10,000–100,000 pg/mL	0.01 pg/mL	Serum	[114]
Sandwich-type electrochemical immunosensor using rGO-TEPA-Thi-Au as a sensitive platform and CMK-3@AuPtNPs as a signal probe	I-T	0.005 to 100 ng/mL	0.0022 ng/mL	Serum	[115]
Monitoring the electrochemical response current of AuPt-vertical graphene/GCE for the oxidation of the methyl orange redox probe	DPV	1 fg/mL to 100 ng/mL	0.7 fg/mL	Serum	[116]
Ordered mesoporous carbon (OMC) doped with AuNPs as a substrate to immobilize AFP antibodies, along with AuPt-MB nanorods as signal probes to bind secondary AFP antibodies and amplify detection	DPV	10 fg/mL to 100 ng/mL	3.33 fg/mL	Serum	[117]
Electrochemical immunosensor based on Fe_3_O_4_NPs@COF-decorated gold nanoparticles and magnetic nanoparticles including SiO_2_@TiO_2_	DPV	0.01 pg/mL to 1 pg/mL	3.30 fg/mL	Serum	[118]
Label-free electrochemical aptasensing using rGO–chitosan–Fc nanocomposites and Au-Pt NPs	DPV	0.001 to 10.0 mg/mL	0.3013 ng/mL	Serum	[119]

## Data Availability

Not applicable.
